# Arboreal route navigation in a Neotropical mammal: energetic implications associated with tree monitoring and landscape attributes

**DOI:** 10.1186/s40462-019-0187-z

**Published:** 2019-12-18

**Authors:** Miguel de Guinea, Alejandro Estrada, K. Anne-Isola Nekaris, Sarie Van Belle

**Affiliations:** 1 Department of Social Sciences, Oxford Brookes University, Gibbs Building, Gipsy Lane, Oxford, OX3 0BP UK; 2 Institute of Biology, National Autonomous University of Mexico, Mexico City, Mexico; 3 Department of Anthropology, University of Texas at Austin, Austin, TX USA

**Keywords:** Landscape, Navigation, Route selection, Cognitive load, Black howler monkey (*Alouatta pigra*), Topological cognitive map, Route-based spatial map

## Abstract

**Background:**

Although navigating along a network of routes might constrain animal movement flexibility, it may be an energetically efficient strategy. Routinely using the same route allows for visually monitoring of food resources, which might reduce the cognitive load and as such facilitate the process of movement decision-making. Similarly, locating routes in areas that avoid costly landscape attributes will enhance their overall energy balance. In this study we determined the benefits of route navigation in an energy minimiser arboreal primate, the black howler monkey (*Alouatta pigra*).

**Methods:**

We monitored five neighbouring groups of black howler monkeys at Palenque National Park, Mexico from September 2016 through August 2017. We recorded the location of the focal group every 20 m and mapped all travel paths to establish a route network (*N* = 1528 travel bouts). We constructed linear mixed models to assess the influence of food resource distribution (*N* = 931 trees) and landscape attributes (slope, elevation and presence of canopy gaps) on the location of routes within a route network.

**Results:**

The number of food trees that fell within the visual detection distance from the route network was higher (mean: 156.1 ± SD 44.9) than randomly simulated locations (mean: 121.9 ± SD 46.4). Similarly, the number of food trees found within the monkey’s visual range per meter travelled increased, on overage, 0.35 ± SE 0.04 trees/m with increasing use of the route. In addition, route segments used at least twice were more likely to occur with increasing density of food resources and decreasing presence of canopy gaps. Route segments used at least four times were more likely to occur in elevated areas within the home ranges but only under conditions of reduced visual access to food resources.

**Conclusions:**

Route navigation emerged as an efficient movement strategy in a group-living arboreal primate. Highly used route segments potentially increased visual access to food resources while avoiding energetically costly landscape features securing foraging success in a tropical rainforest.

## Background

Animal movement strategies are influenced by individuals’ ability to acquire and process information, as well as the energetic requirements of traveling through the landscape [1–3]. Some animal species have been suggested to navigate using cognitive maps: mental representations coding spatio-temporal information of the space where they live in that permits efficient movement decisions [4–8]. Although the elements that construct such spatial representation are still debated [8], the ability to navigate flexibly in space has been suggested to be indicative of sophisticated cognitive maps and, hence, enhanced cognitive abilities [9]. For instance, some animal species have been suggested to compute distances and angles based on features of the landscape as in an Euclidean representation of the area (e.g., honey-bees, *Apis melifera*, [10], African elephants, *Laxodonta africana*, [11], chimpanzees, *Pan troglodytes*, [9]). Contrarily, the most common navigation strategy associated with cognitive maps is the repeated use of paths, here referred to as “habitual routes” (insects [12], birds, [13], mammals, [14, 15], humans, [16]). By following an established set of habitual routes animals and humans benefit from simplifying the decision-making process of movement while still securing foraging success [17].

Even though navigating along habitual routes constrains an animal’s movement flexibility, it may nonetheless enhance its overall energy balance by selecting specific areas along which to navigate that minimize the energetic costs of movement [18]. The accumulation through generations of environmental information within a group of animals can minimise the cost of travelling by optimising the selection of routes to navigate [19]. For instance, pigeons (*Columba livia*) that travelled in pairs increased the linearity of their homing route over the course of multiple generations [19]. Similarly, translocated bighorn sheep (*Ovis canadiensis*) adjusted the location of their migratory routes over time in order to avoid energetically costly areas of the landscape [20]. Combining experiences of several individuals permits foragers not only to efficiently locate resources but also to select the closest or most profitable ones [21]. For example, in ants (*Linepithema humile*), sharing information among group member via pheromone concentration along paths favoured the selection of shorter paths towards food resources [22]. Hence, even though habitual route navigation is frequently hypothesized to be a limiting navigation strategy associated with inferior cognitive skills [6], accumulating and sharing information within groups can lead to the selection of routes that enhance foraging [23] while economising the cost of traveling (avoiding costly landscape features such as mountain ridges [24], and predators, [25]). Additionally, animals with sophisticated cognitive skills might still travel along recurrently used routes because these provide an energetically efficient strategy to navigate [26].

A major potential advantage associated with using habitual route segments is the ability to monitor regularly the phenological states of food resources that are located within animals’ visual range while travelling [18, 27]. Habitual route navigation requires the animal to memorize a series of familiar landmarks (i.e., environmental features) to orientate their movements and relocate feeding and resting sites [28, 29]. The complexity of the cognitive process underlying movement decision-making will increase with the total number of locations to be remembered (e.g., landmarks, food sources; hereafter cognitive load, [30]). Hence, animal’s cognitive load should decrease considerably when only needing to associate multiple food resources with a limited set of travel routes [31, 32]. Reduction in cognitive load may decrease the amount of energy allocated to brain tissue, which in turn can be employed for other physiological processes and/or behavioural activities [33]. Additionally, by clustering the food resources along routes, animals would decrease the energy spent on travelling when searching for food [18].

Similarly, the structure of the area or landscape that is traversed by an animal or a group is undoubtedly linked to the energetic cost of locomotion [34]. Different features of the landscape, such as slope, elevation or substrate, determine the energetic cost of locomotion through an area, which in turn will influence the location of frequently used routes [3, 34]. In terrains with steep slopes, terrestrial animals need to increase their kinetic energy as they move up a slope, increasing the biomechanical and metabolic cost of moving [34, 35]. For instance, Newmark & Rickart [14] showed that wild ungulates (*Odocoileus hemionus* and *Cervus elaphus*) repeatedly used routes that avoided steep slopes to economise energetic expenditure. Likewise, slope at the ground level may influence the movement of animals at the tree level [36]. Arboreal animals were shown to include ground slope into their movement decisions potentially to reduce the cost of travelling, similar to terrestrial animals, assuming a net gain in elevation at the ground is mirrored at the level of tree crowns [37, 38]. In contrast, detectability of further away food resources and neighbouring conspecific groups while travelling may increase in more elevated areas within animal’s home range [18]. Incorporating visual information into movement decisions during travelling likely enhances both terrestrial and arboreal animals’ foraging efficiency and home range defensibility [1, 39].

Lastly, the characteristics of the substrate are important determinants of the energy expenditure for an animals as well [3, 40]. In case of arboreal animals, the characteristics of the substrate, specifically the degree of lateral connectivity, not only influence movement costs but also the availability of substrate itself (i.e., presence of canopy gaps in the forest or deforested areas [41]). McLean et al., [41] demonstrated that crown thickness and density were unifying parameters driving the selection of movement in arboreal animals with different locomotor strategies, which highlights the transversal importance of lateral connectivity in arboreal navigation. Similarly, descending to the ground and re-ascending into trees was shown to increase exponentially the biomechanical costs of movement both in orangutans (*Pongo pygmaeus*, [36]) and human parkour athletes [42]. Optimising the energetic performance during arboreal locomotion likely requires selecting highly inter-connected tree sequences and avoid canopy gaps to navigate [41].

Here, we explore different factors that influence the location of habitual routes of an “energy minimiser” [43], group-living arboreal primate, the black howler monkey (*Alouatta pigra*, hereafter black howlers). The slow transit and long retention times in the digestive system associated with black howlers’ leaf-based diet reduces the availability of metabolic energy [17]. In addition, black howlers engage in highly selective foraging patterns to fulfil their nutritional demands using many different individual trees to forage that vary both within and between years [44]. Memorising all these locations and their respective phenological cycles is assumed to be challenging given the relatively small brain size of these primates [17]. By locating travel routes near potential food resource, black howlers may benefit from reducing their cognitive load by continuously monitoring the status of food resources while travelling [45]. Further, the study was conducted in a terrain where topographic features vary sharply. The influence of elevation changes and occasional gaps of forest coverage could be influencing the movement decisions of black howlers [41, 45]. Hence, the location of habitual routes to navigate is expected to reflect the energy minimising strategy of black howlers by avoiding such costly attributes of the landscape.

First, we hypothesised that black howlers locate their routes along tree sequences that would allow them to visually inspect food resources. We predicted that the number of food resources that fell within the estimated visual range of black howlers would be higher within habitual route segments than at locations outside the route network. Moreover, the number of food resources that can be visually intercepted per metre travelled would increase with a route’s usage frequency. Second, we hypothesised that the location of routes would allow black howlers to avoid costly features of the landscape. We predicted that terrain slope and canopy gaps would negatively influence the occurrence of routes while elevation would have a positive effect only in areas where food trees are less abundant. Overall, we aim to gain insights into the route selection process in arboreal navigation by exploring the benefits associated with travelling frequently along the same tree sequences.

## Materials and methods

### Study site and subjects

We conducted the study in Palenque National Park (PNP, 17°29′N-92°02′W), Mexico, which covers 1171 ha. PNP has a variable terrain due to geological formations and the remains of Mayan ruins underneath the forest floor [46, 47]. As a result, the study area ranges between 65 and 264 m above sea level and the maximum slope of the terrain across the study groups was 41.2 degrees (Table 1). The area covered by canopy gaps (i.e., pasturelands, fallen trees, roads) within the study area was 3.7 ha (7% of the total study area).

**Table 1:** Demographic and environmental summary of the study groups and areas: number of individuals present in each group during the study period; estimated home range size using Kernel 95% estimator; mean number of feeding trees (N FTs) visually detected from the route network till 20 m distance; elevation and slope range within each study group; and, area covered by canopy gaps in hectares and percentages for each study group

Group ID	Group size	HR size (ha)	N FTs visually detected (mean ± SD)	Elevation range (m) (min - max)	Slope range (°) (min - max)	Gaps (ha) (% coverage)
Balam	3–4	10.3	–	170.5–260.0	0.6–39.4	0.3 (3%)
Motiepa	6–8	7.1	161.8 ± 36.9	96.4–173.8	0.3–29.4	0.9 (12.9%)
Naha	5–8	15.3	173.8 ± 35.5	65.0–190.4	0.2–45.2	1.8 (11.9%)
Pakal	7–9	10.6	186.3 ± 38.8	152.6–212.8	0.6–29.4	0.3 (3%)
Unites	4	8.6	102.8 ± 22.5	178.1–264.9	1.5–40.9	0.3 (3.3%)

From September 2016 through August 2017, we observed five groups of wild black howlers, four consecutive days per week (two groups each day) for a total of 297 days and 3104 contact hours (Balam: 58 d, 571 h; Motiepa: 58 d, 628 h; Naha: 63 d, 631 h; Pakal: 61 d, 650 h; Unites: 59 d, 622 h). We designed the data collection protocol such that we sampled the same group only every two or three weeks, leaving a window of one or two weeks in between sampling weeks to control for the productivity of certain tree species.

### Data collection

During observation days (ca. 05:30–17:00), we conducted instantaneous scan samples at 15 min intervals to record the behaviour of all visible group members, as well as the coordinates of the location of the estimated centre of the group using a GPS Garmin 64S (mean GPS error: 6.6 ± SD 2.3 m). In order to obtain detailed information on feeding behaviour, we recorded a feeding bout whenever one or more individuals fed on a plant for a minimum of 5 min accumulated across of feeding individuals (e.g., one individual for 5 min, two individuals each for 3 min, five individuals each for 1 min). For each feeding bout, we recorded the item fed on, the plant species, and at 3 min intervals the number of individuals feeding. We used these feeding bout data to identify the top 10 food species of black howlers at PNP based on the percentage of time spent feeding on each species (*Poulsemia armata*, *Ficus spp.* (6 species), *Brosimum alicastrum*, and *Acacia glomerosa;* see Additional file 1: Table S1). Subsequently, we searched and marked all individual trees (hereafter FTs) with a DBH (diameter at breast height) ≥ 10 cm, including those that groups did not feed on, from these species throughout the home ranges of the study groups (Balam: *N* = 137; Motiepa: *N* = 220; Naha: *N* = 213; Pakal: *N* = 227; Unites: *N* = 134).

We recorded a travel bout whenever two or more group members moved into a neighbouring tree or further until at least two members of the group engaged in a stationary activity different from that in the original tree (i.e., howling, resting or foraging, [48]). In this species, collective group movement is very conspicuous and is typically initiated by an individual leaving the tree and immediately followed by all other group members in a single line progression [48]. Throughout travel bouts, we recorded GPS locations of the estimated group centre every 20 m.

### Data analyses

**Fig. 1:**
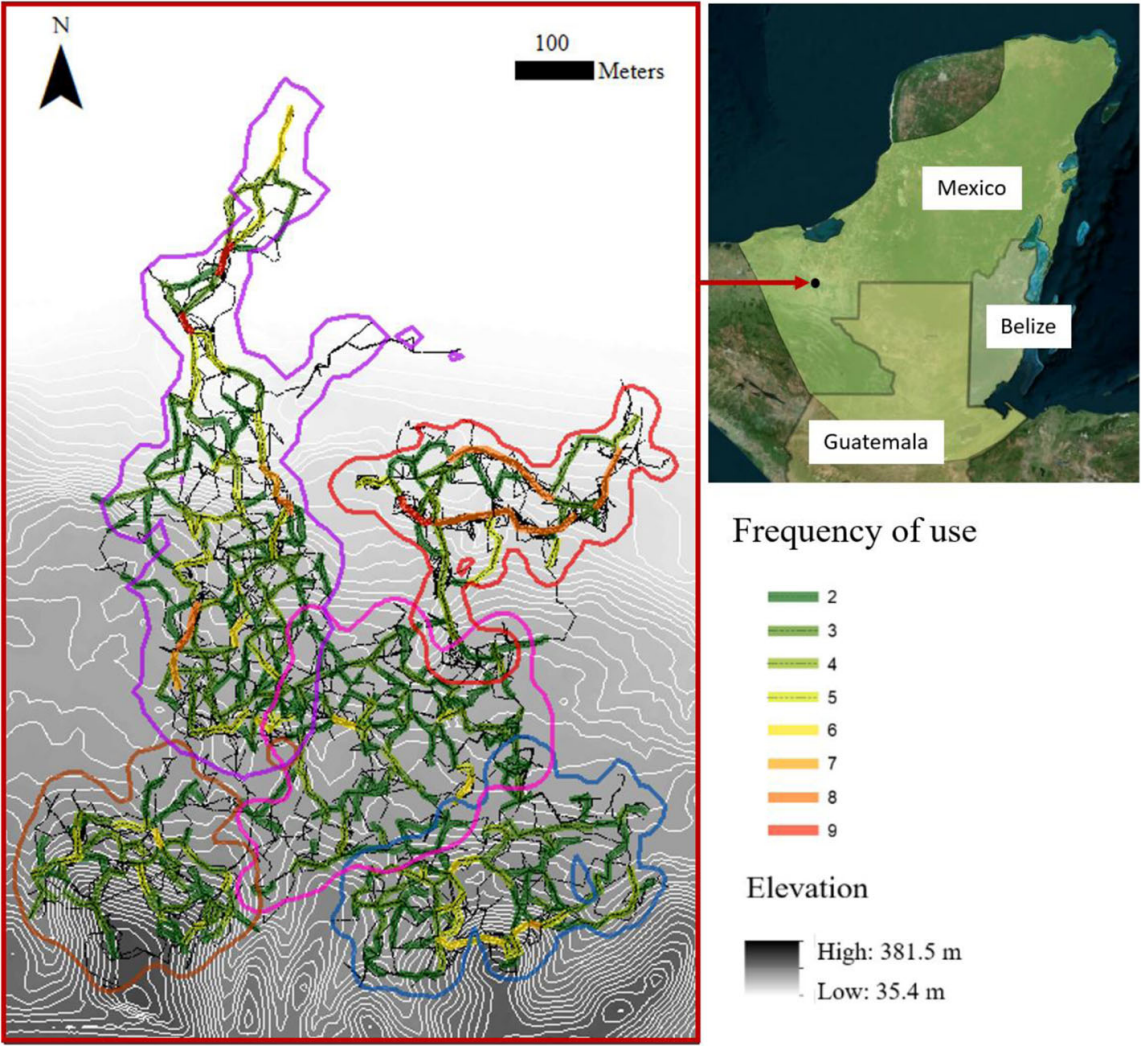
Home ranges (95% kernel density estimates) of five study groups of black howler monkeys at Palenque National Park (Naha: *purple*; Motiepa: *red*; Pakal: *pink*; Balam: *brown*; Unites: *blue*) overlaid on top of elevation contour lines (white lines). Within the home ranges of each study group, we show all recorded travel bouts (black continuous lines), as well as the frequency (green-red scale) that travel segments were used. The geographic distribution of *Alouatta pigra* is shown in the top right of the figure (data from IUCN [50])

We estimated the home range of the study groups using the R package *adehabitatHR* 0.4.15 and the kernel density estimation (KDE) method based on the geographic coordinates recorded during scan samples [49]; Fig. 1). We defined a group’s home range as the 95% KDE isopleth and a core area as the 50% KDE isopleth [48].

In order to construct a habitual route network, we first overlaid all daily travel paths recorded during the same week per group onto a raster map of the area and checked their concordance [18]. Whenever a daily path fell within a 10 m buffer of another daily path of that week for at least 15 m without deviating more than 45° from the other path, we considered it as the same travel segment [11]. We selected these parameters in order to be consistent with previous research [18, 51–53] and to control for GPS accuracy and travel directionality [52, 53]. Food resources in rainforests can occur for a short period in the same location within the same week [54], which can lead to an overestimation of the frequency of used routes [51]. Hence, we first constructed “weekly paths” including only unique daily paths of each sampled week to avoid a bias towards re-used route segments due to short revisiting intervals to certain FTs within that same week. Each group’s weekly paths were overlaid on top of each other and we repeated the same procedure as described above to determine across how many weeks route segments were used [11]. We defined the habitual route network of each group as path segments used during at least two separate observation weeks.

We determined the influence of FT’s distribution on the location of the route networks by simulating the same number of recorded FTs per study group randomly distributed within their respective home ranges, excluding areas with no forest cover [55]. We did not include any cluster parameter into our simulations since most of the tree species were distributed randomly within the study area (*Z*-scores: *Poulsemia armata*, − 3.4; *Ficus spp.*, − 1.9; *Brosimum alicastrum*, − 0.7; and, *Acacia glomerosa*, 0.7; for details see Additional file 1: Table S2, [56]). We ran 10,000 simulations using the R package *rgdal* 1.3–6 [57] and the function “spsample”. Finally, a series of buffers with 5 m increment from 5 m to 20 m were traced around habitual routes of each study group, and the total number of recorded FTs and simulated FTs that fell within each buffer was calculated and statistically compared (see below, [44]). Since we performed 10,000 simulations, we calculated the mean number of simulated FTs that fell within each buffer for each study group to compare it with the number of observed FTs (in Additional file 1: Table S1, we provide the fraction of FTs that fell above the number of simulated location for each buffer). Subsequently, we tested whether the number of FTs that fell within the buffers created along habitual route segments increased with the frequency these routes were reused. For this, we calculated the number of FTs intercepted per meter travelled along each segment to account for segment length.

We determined the topographic attributes of the landscape using an archaeological map of the Mayan city of Palenque [46]. We georeferenced this topographic map, which had a resolution of 4 m, and triangulated the three-dimensional locations into ArcMap 10.4 to create a TIN layer that was converted into a Digital Elevation Model (DEM). We georeferenced the TIN version of the map using 15 salient features of the landscape (e.g., roads, Mayan ruins) visible both on the archaeological map and freely available satellite layers. We overlaid a grid layer of 10 × 10 m^2^ quadrats and extracted values for slope and elevation for each quadrat using the Spatial Analysist tool from ArcMap 10.4. For elevation, we determined the height at the centre of each quadrat. For slope, we calculated the maximum rate of change in elevation for each quadrat relative to its adjacent quadrats [58]. We marked the edges of canopy gaps in the field using a GPS device and corrected them using satellite imagery. We used remotely sensed images on land cover from NASA’s Landsat 8 satellite for this purpose [59]. Subsequently we created a buffer of 25 m from the centre of each quadrat and calculated the percentage overlap between such a buffer and the recorded canopy gaps.

We determined the potential of visual inspection of FTs while travelling through habitual route segments by creating a series of buffers every 5 m from the centre of each quadrat up till 35 m, which is the estimated visual detection distance of howlers [17, 45]. We counted all marked FTs contained within each buffer including the FTs already counted in small buffers (e.g., when counting FTs contained within the 10 m buffer, we counted those contained within the 5 m buffer again). Subsequently, we divided the number of FTs contained in each buffer by the total number of FTs within the home range of the study group. We calculated these for the preferred tree species within each home range separately. Finally, per quadrat, values for all buffers were summed and divided by the number of buffers (*N* = 7). By including previously counted trees in smaller buffers into large buffers, we emphasised the importance of FTs near the centre of the cell. Thus, we calculated an index of FT’s density near the centre of each quadrat as a measure for visual access to potential food resources (hereafter called “FTs density”; see Additional file 2: Figure S1; and, Additional file 3: Figure S2).

### Statistical analysis

We conducted all statistical models in R 3.5.2 (R Core Team, 2018) and implemented them using the functions *lmer* and *glmer* of the *lme4* package 1.1–21 [60]. We fitted two LMMs to determine the influence of FTs on the location of routes used by black howlers.

First, we tested whether routes intercepted more FTs than by chance by using the number of locations intercepted per buffer around the route network as response variable and type of location (real or mean of simulated FTs) as predictor variable [model 1: N FTs ~ type of location + buffer size + (1 + type of location + buffer size| group ID)]. For the second model, the response variable was the number of FTs intercepted per meter travelled and the frequency of use of a certain route segment was the predictor variable [model 2: *log*(N FTs per metre) ~ frequency of use of the segment + buffer size + (1 + frequency of use of the segment + buffer size | group ID)]. For both models, buffer size was used as a control variable since we would expect that larger buffers may intercept a larger number of FTs. We excluded one study group (Balam) from these two models because its composition and home range location changed in January 2017 [61].

In addition, we fitted two GLMMs with a binomial error structure and a logit link function instead of one with continuous outcome to test the influence of landscape features on the location of route segments. For these models, the response variable was the presence/absence of routes for each quadrat, either used at least twice or at least four times, respectively. Previous research highlighted that the characteristics of routes used at least twice and at least four times differed from each other [51]. Thus, we decided to construct separate models. For both models, the predictor variables were elevation, slope, percentage of area covered by gaps, and FTs density per quadrat. We included an interaction between elevation and FTs density in both GLMMs since we predicted that black howlers located routes to navigate in more elevated areas only when the visual access to food resources decreased.

To account for potential differences among groups, we included group ID as a random variable (random intercept) in all models. In order to allow for fixed effect predictors to vary among the levels of the random effect variables, random slope terms were included in the models as well. In addition, we incorporated two control variables: intergroup overlap (whether a certain quadrat was used by multiple groups or not) and home range location (whether the quadrat fell inside or outside the group’s core area).

We controlled for spatial autocorrelation by determining an autocorrelation term from the full model and subsequently including this term as a control variable in a newly fitted full model [62]. These terms were calculated as the average of the residuals from the original model (for all data points from the same group) weighted by the distance to the particular data point. The weight followed a normal distribution for which the standard deviation (*D*) was optimised such that the log-likelihood of the full model including the autocorrelation term was maximised (here: *D* = 5–7; based on [62]) [model 3: presence of route ~ *sqrt* (slope) + presence of gap + elevation * FTs density + overlap + area (home range or core) + autocorrelation term + (1 + *sqrt* (slope) + presence of gap + elevation + FTs density + overlap + area + autocorrelation term + elevation: FTs density | | group ID)].

We verified that the residuals of the models were normally and homogeneously distributed by visually inspecting qq-plots and plotting them against fitted values. We also tested for multicollinearity by inspecting variance inflation factors using the *vif* function from the *car* package [63]. Similarly, we assessed the stability of the GLMMs by comparing the estimates derived from a model based on all data with those obtained from models based on subset which excluded levels of the random effects one at a time. No model assumptions were violated. In all cases, we compared the full model to a corresponding null model (with only random and control variables) using likelihood-ratio tests (*anova* function set to “Chisq”). When an interaction term had no significant effect, we ran a reduced model including only the main effects. Finally, if the likelihood-ratio test for full and null model comparison was significant, we inspected the significance of each predictor variable using likelihood- ratio tests comparing full models with reduced models without the variables of interest, using the *drop1* function [64].

## Results

We recorded a total of 1528 travel bouts (mean: 305.6 ± SD 43.9 travel bouts per group). On average, the length of an individual travel bout was 65.3 ± SD 57.5 m and the daily path length was 365.8 ± SD 199.2 m, ranging from 28.2 m to 1022.8 m. Between 64.5 and 75.1% of the travel bouts fell within the habitual route network (paths used at least twice), which had a mean length of 3.2 ± SD 1.2 km per group (Fig. 1). Home range size ranged between 7.1–15.3 ha while the relative difference in elevation within each study group was 86.2 ± SD 24.8 m (Table 1).

### Spatial distribution of food trees

**Fig. 2:**
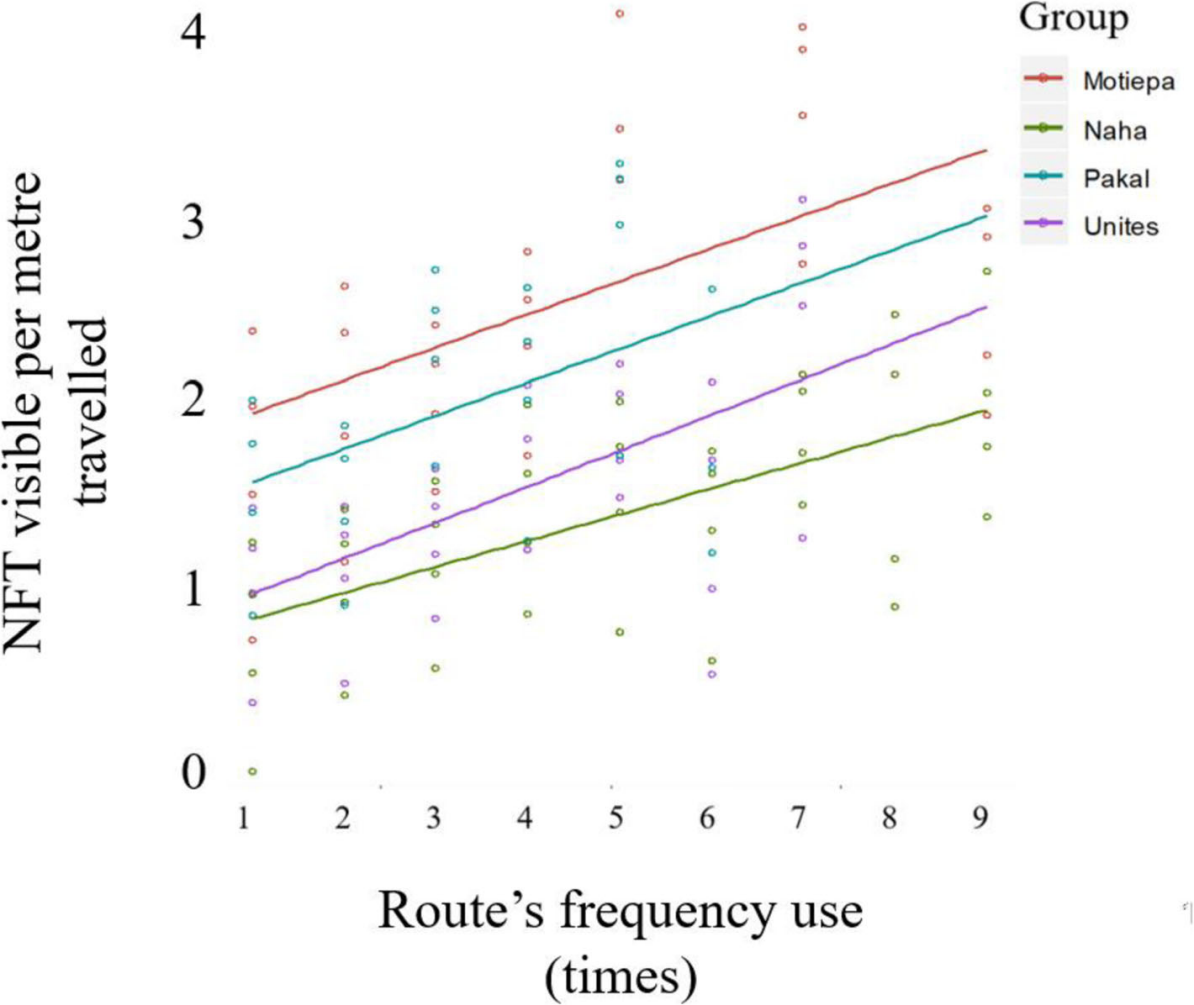
Fitted linear regression of number of feeding trees (FTs) intercepted per metre travelled against frequency of use of route’s segments. The plot shows the four study groups that were included in this analysis. Note that route segments used once were travel bouts that never overlapped other travel bouts

The total number of FTs within the assumed visible detection range from the habitual route network (mean 156.1 ± SD 44.1 trees per group) was significantly higher than would be expected by chance (mean 122.0 ± SD 46.4 locations) as shown by the likelihood ratio test (*χ*^*2*^ = 7.9, Estimate = − 34.2 ± 6.9, df = 1, *P* = 0.005). Similarly, the number of FTs potentially visible from the habitual route network per metre travelled increased with increasing frequency of use of the segment (likelihood ratio test: *χ*^*2*^ = 15.1, Estimate = 0.4 ± 0.04, df = 1, *P* <  0.001). For instance, the number of FTs that fell within the estimated visual range along route segments used twice was 0.07 ± 0.04 trees/m while it increased to 0.11 ± 0.05 trees/m in segments used four times and to 0.24 ± 0.20 in segments used seven times (Fig. 2).

### Landscape attributes

We found that the GLMMs for routes used at least twice and routes used at least four times provided a significantly better fit than the null models (Table 2). The probability of a quadrat containing a route that was used at least twice increased with FTs density but decreased with presence of canopy gaps. After removing a non-significant interaction from the full model (see Additional file 1: Table S1 for full model results), we did not find a significant influence of either elevation or slope on the probability of a quadrat containing a route used at least twice.

**Table 2:** Results of two GLMMs testing the influence of different landscape attributes (slope, presence of canopy gaps, elevation and FTs density) on the probability occurrence of a route segment used at least twice and four times within a certain quadrat. Group ID (*N* = 5) was included as random effect in the model. We compared the full model to a corresponding null model (with only random and control variables) using likelihood-ratio tests. All *p*-values < 0.05 are shown in bold for clarity

Response variable	Probability of locating a route used at least twice					Probability of locating a route used at least four times				
Full null model comparison	χ2 = 23.2, d.f. = 5, *p* < 0.001					χ2 = 18.8, d.f. = 5, *p* = 0.002				
Predictor variable	Est.	s.e.	CI_lower_	CI_upper_	*p*-value	Est.	s.e.	CI_lower_	CI_upper_	*p*-value
(Intercept)	−0.156	0.574	−1.567	1.209	a	− 3.126	0.298	−6.359	−2.531	a
Slope	0.139	0.104	−0.128	0.481	0.182	0.390	0.139	0.006	0.752	0.058
Presence of canopy gaps	−0.803	0.115	−1.088	−0.482	**0.001**	−0.354	0.186	−0.452	0.023	0.128
Elevation	0.446	0.328	−0.323	1.298	0.198	0.944	0.550	−0.363	2.237	0.173
FTs density	0.896	0.213	0.372	1.390	**0.006**	0.967	0.246	0.401	1.533	**0.010**
Elevation * FTs density	−0.280	0.210	−0.783	0.340	0.273	−0.439	0.140	−0.809	−0.374	**0.041**
Overlapping area ^b^	0.274	0.136	−0.014	0.689	0.054	−1.000	0.203	−3.398	−0.437	**0.003**
Location within the HR ^b^	−0.538	0.442	−1.609	0.518	0.255	−0.724	0.499	−1.414	0.838	0.190
Autocorrelation term ^b^	2.487	0.072	2.347	2.632	**< 0.000**	2.943	0.100	1.636	3.252	**< 0.000**

^a^Not shown because of having no meaningful or very limited interpretation

^b^Represent control predictors included in the model

**Fig. 3:**
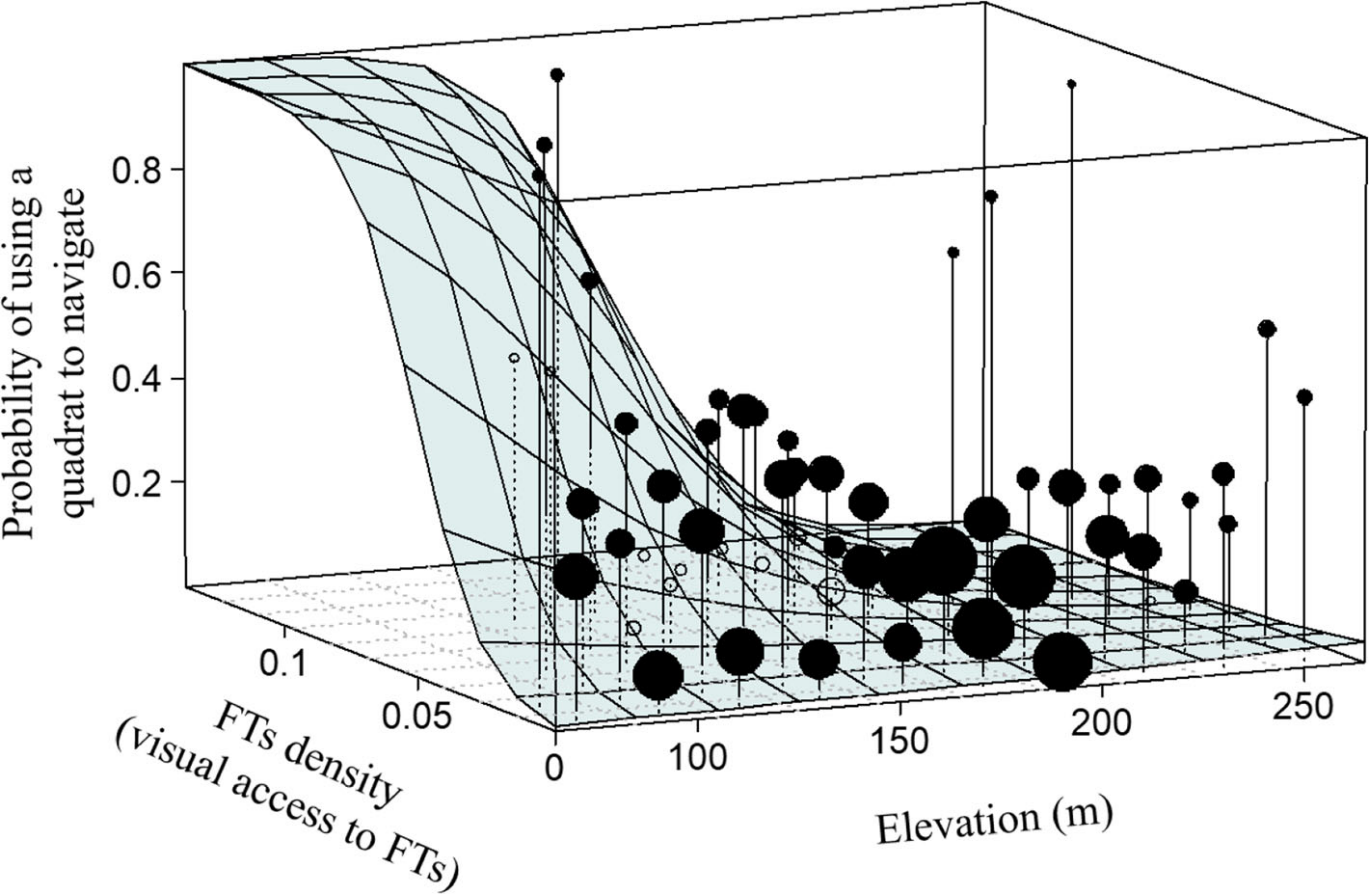
Probability that black howler monkeys selected a quadrat to navigate at least in four different occasions in relation to the relative elevation within their home range and FTs density (proxy for visual access to FTs). The height of spheres represents the probability that a certain quadrat was chosen to navigate per combination of elevation and FTs density. Each surface (i.e., square) represents the expected probability of a quadrat to be chosen according to the model (conditional on all other predictors being set at their average value). Sphere size corresponds to the relative number of observations, with closed circles being above the model surface and open circles below

Similar to the previous model, routes used at least four times were significantly influenced by FTs density. Contrary to the previous model, there was not a significant influence of gap presence on the location of a route used at least four times. There was a significant effect of the interaction between elevation and FTs density in the model (Fig. 3). As we predicted, there was a positive effect of elevation only in areas with potentially low visual access to FTs. Even though the effect of slope in the model was not significant, there was a positive trend towards locating routes used at least four times in quadrats with highly pronounced slopes (Table 2).

## Discussion

Our findings show that black howlers navigate along a habitual route network linked to the distribution of potential food resources and landscape attributes. While using specific routes to navigate limits an animal’s movement options [65], black howlers seem to counteract such constraints by optimising the location of their habitual routes. Such knowledge, potentially accumulated over generations, may lead to the selection of routes that minimises energy expenditure and favours tree monitoring while traveling [1, 66].

Because of the logistical difficulties of observing monitoring behavior in arboreal animals (i.e., turning their head combined with fixed gaze towards food resources while travelling, [27]), we developed a method to infer the probability that arboreal animals monitored FTs while travelling through habitual routes. Such a measure considers the density of FTs nearby frequently used routes, and thus the potential visual access of those FTs. Our findings show that black howlers at PNP intercept a higher number of food resources within their visual range while travelling along their habitual route networks than they would be expected by chance. That is, the number of food resources visually intercepted increased 19% with the number of times a certain route segment was used. These results are consistent with the hypothesis that black howlers use habitual route networks to facilitate the visual inspection of potential food resources [17, 45].

While travelling through areas with high FTs densities facilitates the potential monitoring of FTs, black howlers could also be enhancing their foraging success by gaining access to FTs. However, the high reusage frequency of some route segments makes it unlikely that food was always available every time they travelled along that specific route. Furthermore, we did not only include in the analyses trees in which we observed monkeys feed but all trees from preferred tree species present in the study area from which they did not feed during our observations. Therefore, black howlers potentially combined increasing accessibility and monitoring of FTs while travelling along route segments. By securing foraging success while reducing the number of locations that need to be memorised, the complexity of processing movement decisions decreases [65, 67]. In addition, a reduction in the number of operative elements to include in the cognitive processes enhances the planning abilities of animals and humans [39, 68]. Specifically, arboreal primates have been shown to plan future movements using information obtained from monitoring feeding trees while travelling [69] or to re-direct their trajectories towards specific tree species after inspecting other trees from the same species [69, 70]. By gathering ecological information on patterns of food availability, animals increase the predictability of a successful foraging event across heterogeneous landscapes [67].

Black howlers navigated through elevated areas only under decreasing FTs density on route segments used on four different occasions or more (see Additional file 4: Figure S3). Likely, elevated areas increased black howlers’ visual access over the landscape enhancing the detectability of faraway food resources. Arboreal primates in rainforests need to find strategies to enhance their visual window typically obstructed by foliage, tree trunks or lianas [71]. Here, we provide evidence for the first time that supports the use of elevated areas as a strategy to enhance information acquisition via visual cues of food resources, which was suggested in previous studies on arboreal primate navigation [38, 45, 72]. Spiegel & Crofoot [1] asserted that not only the information state of an individual will determine its movement patterns but also the acquisition of information while “on the move”. The attention black howlers allocated to search in their environment likely increased by following habitual routes and selecting suitable terrain to navigate increasing the acquisition of information and enhancing their movement decisions [73]. Subsequent research should address adjustment in the spatial performance of animals following enhanced visual or olfactory cues *en route*.

Even though we predicted that black howlers at PNP would avoid slopes to economise the expenditure of energy, we found a positive trend on the selection of routes associated with increased slope. The energetic cost of travelling along slopes for a large bodied animal represents a major challenge at ground level [74] but it may not be the case in the trees. Contrarily, travelling through sharp slopes may increase visual access over the landscape as discussed above [38]. Hence, patterns found in terrestrial animals cannot be assumed in arboreal animals since arboreality may impose other energetic challenges [74]. Howlers’ main locomotor strategy is quadrupedalism [75], which means that moving vertically (descending to the ground and re-ascending to the canopy) rather than horizontally increases the number and complexity of movements in which they engage [41]. Thus, selecting areas with a continuous horizontal substrate (i.e., the canopy) might be more energetically advantageous than avoiding a terrain’s ground slopes [76]. Indeed, our results show a tendency of selecting areas in which to navigate that avoid proximity to gaps in the canopy, similar to other arboreal animals [41, 45]. By avoiding canopy gaps, black howlers improve their overall energetic balance facilitating their locomotion but may also reduce their exposure to pathogens and terrestrial predators [76].

Overall, black howlers’ navigation behaviour suggests that the current location of their habitual route network is the result of an optimization process to counteract the effect of different landscape attributes. Jang et al. [16] argued that the low visibility and widely distributed food sources of rainforests might have driven the development of extensive spatial knowledge and memory of food locations both in chimpanzees and human hunter-gatherers. Similarly, black howlers evolved strategies to enhance their visibility in rainforests and ease the acquisition of food resources. We argue that the accumulation of information through generations within social units could provide black howlers a strategy to progressively adjust the location of their routes to engage in efficient movement patterns [1]. Here, we provide evidence that efficient navigation can be achieved by travelling through habitual route networks but it is essential that future research addresses the optimisation process leading route’s location to fully understand the role of information accumulation and sharing in movement ecology.

## Conclusions

Route networks are advantageous mechanisms for arboreal animals to navigate and locate food resources in rainforests. Black howler's route network at our field site allowed them to visually intercept a high number of food resources while travelling, which potentially reduced their cognitive load and facilitated resource monitoring. Similarly, the importance of visual access to food resources was also shown by the tendency of black howler monkeys to travel through highly used route segments in elevated areas only under conditions of limited visual access to food resources. Arboreality imposed howlers to travel through areas void of canopy gaps, which potentially reduced the energetic costs of crossing gaps. The present study is the first fine-scale analyses addressing the influence of landscape features on arboreal animal movement patterns. Due to the dynamic nature of rainforests, future studies should consider the temporal component of route networks which may potentially mirror intra- and interannual fluctuations of rainforests.

## Supplementary information

**Additional file 1: Table S1.** Total feeding time and overall percentage of time spent by black howler monkeys on the top ten tree species at Palenque National Park. **Table S2.** Clustering analyses to determine the spatial patterns of black howler monkey’s preferred tree species. **Table S3.** The comparison between the number of real FTs and simulated locations that fell within a series of buffers between 5 and 20 metres traced from the route networks of the study groups was always significant. **Table S4.** Results of the LMM testing differences among the number of locations computationally simulated and marked FT that fell within the visual detection distance of black howler monkeys. **Table S5.** Results of the LMM testing for differences in the number of FTs visually intercepted per meter travelled along route segments with different usage frequency (e.g., twice, 3 times, … until 9 times). **Table S6**. Results of the full GLMM testing the influence of different landscape attributes. **Table S7.** Random slopes and estimated variance components (standard deviations) for the random effects and residuals from the model testing the influence of landscape attributes on the occurrence of routes used at least twice within a quadrant. **Table S8**. Results of the full GLMM testing the influence of different landscape attributes (slope, presence of canopy gaps, elevation and visibility of feeding trees^1^) on the occurrence of a route segment used at least four times within a certain quadrat. **Table S9.** Random slopes and estimated variance components (standard deviations) for the random effects and residuals from the model testing the influence of landscape attributes on the occurrence of routes used at least four times within a quadrant.

**Additional file 2: Figure S1.** Graphic description of the method used to estimate black howler monkey's potential visibility of food resources throughout their home range.

**Additional file 3: Figure S2.** Three-dimensional representation of Palenque National Park and the study groups of black howler monkeys.

**Additional file 4: Figure S3.** Probability that black howler monkeys selected a quadrat to navigate in between three and height different occasions in relation to the relative elevation within their home range and the visibility of FT.

## Data Availability

The datasets used and/or analysed during the current study are available from the corresponding author on reasonable request.

## Abbreviations

d: Days
DEM: Digital Elevation Model
FTs: Feeding Trees
GLMM: Generalized Linear Mixed Model
h: Hours
ha: Hectares
KDE: Kernel Density Estimator
m: Metres
PNP: Palenque National Park
SD: Standard deviation
SE: Standard error
