# Supplementary material for: Arboreal route navigation in a Neotropical mammal: energetic implications associated with tree monitoring and landscape attributes
Source: Mov Ecol. 2019 Dec 18;7:39. doi: 10.1186/s40462-019-0187-z (PMC6918719; doi:10.1186/s40462-019-0187-z)
Supplement: Supplementary file 1 — Additional file 1: Table S1. Total feeding time and overall percentage of time spent by black howler monkeys on the top ten tree species at Palenque National Park. Table S2. Clustering analyses to determine the spatial patterns of black howler monkey’s preferred tree species. Table S3. The comparison between the number of real FTs and simulated locations that fell within a series of buffers between 5 and 20 metres traced from the route networks of the study groups was always significant. Table S4. Results of the LMM testing differences among the number of locations computationally simulated and marked FT that fell within the visual detection distance of black howler monkeys. Table S5. Results of the LMM testing for differences in the number of FTs visually intercepted per meter travelled along route segments with different usage frequency (e.g., twice, 3 times, … until 9 times). Table S6. Results of the full GLMM testing the influence of different landscape attributes. Table S7. Random slopes and estimated variance components (standard deviations) for the random effects and residuals from the model testing the influence of landscape attributes on the occurrence of routes used at least twice within a quadrant. Table S8. Results of the full GLMM testing the influence of different landscape attributes (slope, presence of canopy gaps, elevation and visibility of feeding trees1) on the occurrence of a route segment used at least four times within a certain quadrat. Table S9. Random slopes and estimated variance components (standard deviations) for the random effects and residuals from the model testing the influence of landscape attributes on the occurrence of routes used at least four times within a quadrant. [file 40462_2019_187_MOESM1_ESM.docx]

| **Table S1.** Total feeding time and overall percentage of time spent by black howler monkeys on the top ten tree species at Palenque National Park. | | |
| --- | --- | --- |
| **Species** | **Time (*min*)** | **Percentage** |
| *Poulsenia armata* | 8824 | 14.8 |
| *Ficus yoponensis* | 8342 | 14.0 |
| *Brosimum alicastrum* | 4962 | 8.4 |
| *Ficus aurea* | 2946 | 4.9 |
| *Ficus americana* | 2929 | 4.9 |
| *Acacia glomerosa* | 2891 | 4.8 |
| *Ficus insipida* | 2738 | 4.6 |
| *Ficus maxima* | 2323 | 3.9 |
| *Ficus crassinervia* | 1559 | 2.6 |
| *Ficus pertusa* | 1521 | 2.6 |

| **Table S2.** Clustering analyses to determine the spatial patterns of black howler monkey’s preferred tree species. The nearest neighbour analysis examines the distance between each FT and the closest FT to it, and then compares such distance to expected values of a random sample of points. The nearest neighbour index (NNI) is the sum of all distances divided by the total number of points (1 = random distribution; >1 = cluster distribution; and, <1 = dispersed distribution). A negative Z-score indicates clustering while a positive Z-score indicates dispersion or evenness. | | | | | |
| --- | --- | --- | --- | --- | --- |
| **Tree_sp** | **Group** | **Observed distance (m)** | **Expected distance (m)** | **NN index** | **Z-Score** |
| *Acacia* | Motiepa | 16.6 | 23.9 | 0.70 | -2.95 |
|  | Naha | 67.5 | 56.1 | 1.12 | 1.23 |
|  | Pakal | 143.2 | 68.8 | 2.08 | 4.63* |
|  | Unites | 28.2 | 27.9 | 1.01 | 0.06 |
| *Brosimum alicastrum* | Motiepa | 30.9 | 28.1 | 1.16 | 1.38 |
|  | Naha | 16.2 | 20.7 | 0.78 | -2.76 |
|  | Pakal | 16.0 | 19.3 | 0.83 | -2.72 |
|  | Unites | 16.1 | 17.6 | 0.96 | -0.61 |
| *Ficus spp.* | Motiepa | 16.2 | 23.1 | 0.70 | -2.91 |
|  | Naha | 22.6 | 29.4 | 0.76 | -3.13 |
|  | Pakal | 21.0 | 24.6 | 0.85 | -1.93 |
|  | Unites | 27.9 | 27.1 | 1.03 | 0.32 |
| *Poulsemia armata* | Motiepa | 10.1 | 14.9 | 0.72 | -5.91* |
|  | Naha | 14.6 | 23.3 | 0.62 | -6.75* |
|  | Pakal | 13.4 | 15.3 | 0.88 | -2.17 |
|  | Unites | 29.0 | 25.9 | 1.12 | 1.07 |
| significant* | | | | | |

**Table S3.** The comparison between the number of real FTs and simulated locations that fell within a series of buffers between 5 and 20 metres traced from the route networks of the study groups was always significant. The number of FTs that fell within the buffers was always between 2.15 and 9.6 standard deviations higher than the number of simulated locations.

| **Group** | **Buffer (m)** | **N Real FTs** | **N simulated locations** | ***z*-score** | ***p*-value** |
| --- | --- | --- | --- | --- | --- |
| *Motiepa* | 5 | 113 | 66 | 6.90 | <0.001 |
|  | 10 | 154 | 120 | 4.67 | <0.001 |
|  | 15 | 184 | 159 | 3.82 | <0.001 |
|  | 20 | 196 | 184 | 2.15 | 0.016 |
| *Naha* | 5 | 123 | 75 | 6.95 | <0.001 |
|  | 10 | 177 | 129 | 6.71 | <0.001 |
|  | 15 | 192 | 161 | 5.04 | <0.001 |
|  | 20 | 203 | 178 | 4.66 | <0.001 |
| *Pakal* | 5 | 132 | 66 | 9.60 | <0.001 |
|  | 10 | 186 | 121 | 8.66 | <0.001 |
|  | 15 | 207 | 188 | 3.40 | <0.001 |
|  | 20 | 220 | 160 | 8.67 | <0.001 |
| *Unites* | 5 | 73 | 45 | 5.29 | <0.001 |
|  | 10 | 99 | 79 | 3.44 | <0.001 |
|  | 15 | 114 | 103 | 2.35 | 0.009 |
|  | 20 | 125 | 118 | 1.88 | 0.029 |

**Table S4.** Results of the LMM testing differences among the number of locations computationally simulated and marked FT that fell within the visual detection distance of black howler monkeys.

| **Predictor variable** | **Est.** | **s.e.** | | ***p*-value** | |
| --- | --- | --- | --- | --- | --- |
| (Intercept) | 150.2 | 16.4 | | ^a^ | |
| Type of location  (FT or simulated location) | -34.2 | 6.9 | | **< 0.001** | |
| Buffer size | 30.3 | 5.6 | | **< 0.001** | |
| **Random effect** | **Term^1^** | | **Standard deviation** | |  |
| Group ID | (Intercept) | | 31.58 | |  |
| Group ID | Type of location  (FT or simulated lcoation) | | 11.44 | |  |
| Number of observations = 32; number of levels of random effects: Group IDs = 4  ^1^ The column ‘term’ specifies whether the row refers to a random intercept or random slope component. | | | | |  |

**Table S5.** Results of the LMM testing for differences in the number of FTs visually intercepted per meter travelled along route segments with different usage frequency (e.g.. twice. 3 times. … until 9 times).

| **Predictor variable** | **Est.** | **s.e.** | | ***p*-value** | |
| --- | --- | --- | --- | --- | --- |
| (Intercept) | -2.43 | 0.22 | | ^a^ | |
| Times used | 0.35 | 0.04 | | **< 0.001** | |
| Buffer size | 0.36 | 0.92 | | **< 0.001** | |
| **Random effect** | **Term^1^** | | **Standard deviation** | |  |
| Group ID | (Intercept) | | 0.42 | |  |
| Group ID | Times used | | 0.03 | |  |
| Group ID | Buffer 5 m | | 0.04 | |  |
| Group ID | Buffer 15 m | | 0.02 | |  |
| Group ID | Buffer 20 m | | 0.00 | |  |
| Number of observations = 116; number of levels of random effects: Group IDs = 4  ^1^ The column ‘term’ specifies whether the row refers to a random intercept or random slope component. | | | | |  |

**Table S6**. Results of the full GLMM testing the influence of different landscape attributes (slope. presence of canopy gaps. elevation and visibility of feeding trees^1^) on the occurrence of a route segment used at least twice within a certain quadrat.

| **Predictor variable** | **Est.** | **s.e.** | ***p*-value** |
| --- | --- | --- | --- |
| (Intercept) | -31.26 | 0.29 | ^a^ |
| Slope | 0.39 | 0.14 | 0.005 |
| Presence of canopy gaps | 0.35 | 0.19 | 0.057 |
| Elevation | -0.26 | 0.12 | 0.032 |
| Visibility of FT^1^ | -0.59 | 0.11 | < 0.001 |
| Elevation * Visibility of FT^1^ | -0.44 | 0.14 | 0.002 |
| Overlapping area ^b^ | -0.74 | 0.20 | < 0.001 |
| Location within the HR ^b^ | -0.72 | 0.50 | 0.147 |
| Autocorrelation term ^b^ | 29.43 | 0.10 | < 0.001 |
| ^a^ Not shown because of having no meaningful or very limited interpretation. | | | |
| ^b^ Represent control predictors included in the model | | | |
|  | | | |

**Table S7.** Random slopes and estimated variance components (standard deviations) for the random effects and residuals from the model testing the influence of landscape attributes on the occurrence of routes used at least twice within a quadrant.

| **Random Effect** | **Term^1^** | **Standard deviation** |
| --- | --- | --- |
| Group ID | (Intercept) | 1.25 |
| Group ID | Slope | 0.00 |
| Group ID | Presence of canopy gaps | 0.16 |
| Group ID | Elevation | 0.67 |
| Group ID | Visibility of FT | 0.41 |
| Group ID | Elevation * Visibility of FT | 0.35 |
| Group ID | Overlapping area | 0.00 |
| Group ID | Location within the HR | 0.95 |
| Group ID | Autocorrelation term | 0.00 |
| Number of observations = 5037; number of levels of random effects: Group IDs = 5  ^1^ The column ‘term’ specifies whether the row refers to a random intercept or random slope component. | | |

**Table S8**. Results of the full GLMM testing the influence of different landscape attributes (slope. presence of canopy gaps. elevation and visibility of feeding trees^1^) on the occurrence of a route segment used at least four times within a certain quadrat.

| **Predictor variable** | **Est.** | **s.e.** | ***p*-value** |
| --- | --- | --- | --- |
| (Intercept) | -31.26 | 0.29 | ^a^ |
| Slope | 0.39 | 0.14 | 0.005 |
| Presence of canopy gaps | 0.35 | 0.19 | 0.057 |
| Elevation | -0.26 | 0.12 | 0.032 |
| Visibility of FT^1^ | -0.59 | 0.11 | < 0.001 |
| Elevation * Visibility of FT^1^ | -0.44 | 0.14 | 0.002 |
| Overlapping area ^b^ | -0.74 | 0.20 | < 0.001 |
| Location within the HR ^b^ | -0.72 | 0.50 | 0.147 |
| Autocorrelation term ^b^ | 29.43 | 0.10 | < 0.001 |
| ^a^ Not shown because of having no meaningful or very limited interpretation. | | | |
| ^b^ Represent control predictors included in the model | | | |
|  | | | |

**Table S9.** Random slopes and estimated variance components (standard deviations) for the random effects and residuals from the model testing the influence of landscape attributes on the occurrence of routes used at least four times within a quadrant.

| **Random Effect** | **Term^1^** | **Standard deviation** |
| --- | --- | --- |
| Group ID | (Intercept) | 0.70 |
| Group ID | Slope | 0.00 |
| Group ID | Presence of canopy gaps | 0.24 |
| Group ID | Elevation | 0.00 |
| Group ID | Visibility of FT | 0.00 |
| Group ID | Elevation * Visibility of FT | 0.22 |
| Group ID | Overlapping area | 0.00 |
| Group ID | Location within the HR | 1.05 |
| Group ID | Autocorrelation term | 0.00 |
| Number of observations = 5037; number of levels of random effects: Group IDs = 5  ^1^ The column ‘term’ specifies whether the row refers to a random intercept or random slope component. | | |
