# Supplementary material for: Arboreal route navigation in a Neotropical mammal: energetic implications associated with tree monitoring and landscape attributes
Source: Mov Ecol. 2019 Dec 18;7:39. doi: 10.1186/s40462-019-0187-z (PMC6918719; doi:10.1186/s40462-019-0187-z)
Supplement: Supplementary file 2 — Additional file 2: Figure S1. Graphic description of the method used to estimate black howler monkey's potential visibility of food resources throughout their home range. [file 40462_2019_187_MOESM2_ESM.docx]

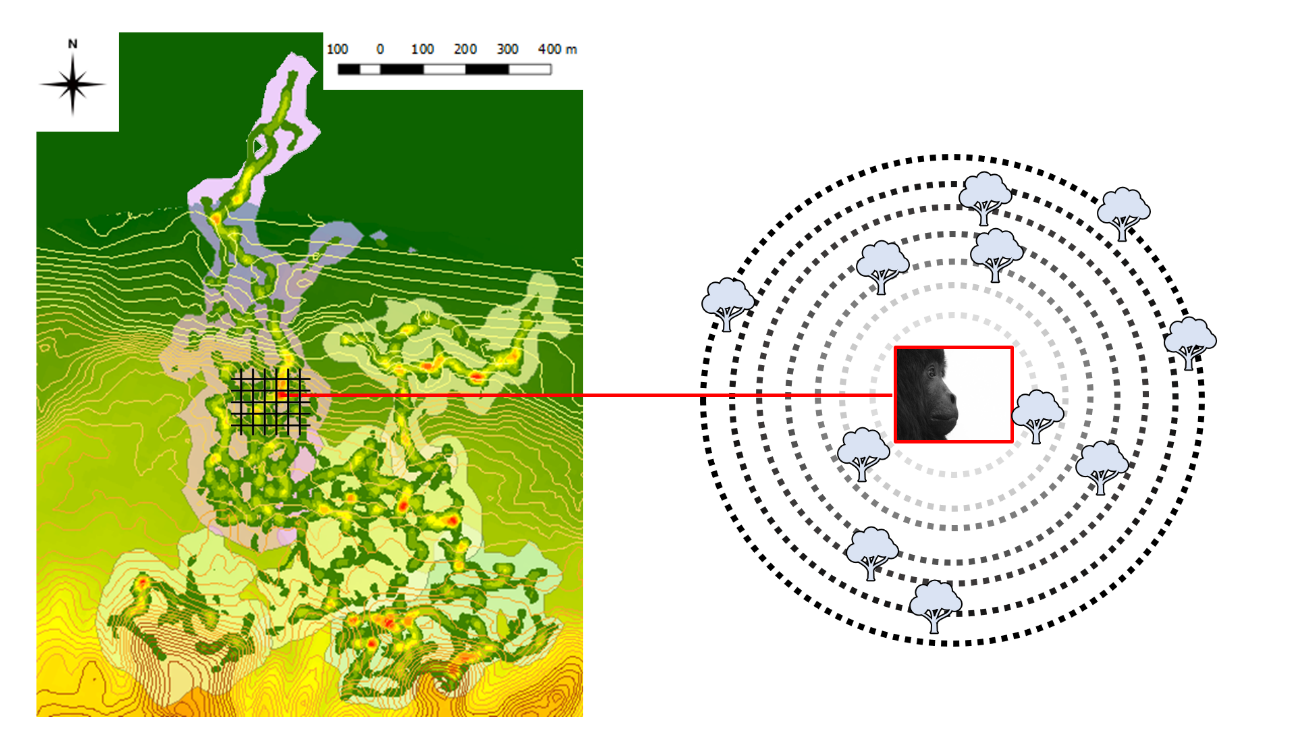


**Fig. S1** Map showing the home ranges (Kernel estimate at 95%) of the study groups of Black howler monkeys overlaid on top of the contour lines and the terrain’s elevation. The frequency of use of the route network is illustrated from dark green (low) to red (high). A grid-cell of 10 x 10m^2^ was overlaid and the landscape attributes of each cell were extracted. A series of buffers from 5-35 m were traced from the centre of each cell to determine the visibility ability of the monkeys from every location within their home range.
