# Supplementary material for: Arboreal route navigation in a Neotropical mammal: energetic implications associated with tree monitoring and landscape attributes
Source: Mov Ecol. 2019 Dec 18;7:39. doi: 10.1186/s40462-019-0187-z (PMC6918719; doi:10.1186/s40462-019-0187-z)
Supplement: Supplementary file 4 — Additional file 4: Figure S3. Probability that black howler monkeys selected a quadrat to navigate in between three and height different occasions in relation to the relative elevation within their home range and the visibility of FT. [file 40462_2019_187_MOESM4_ESM.docx]

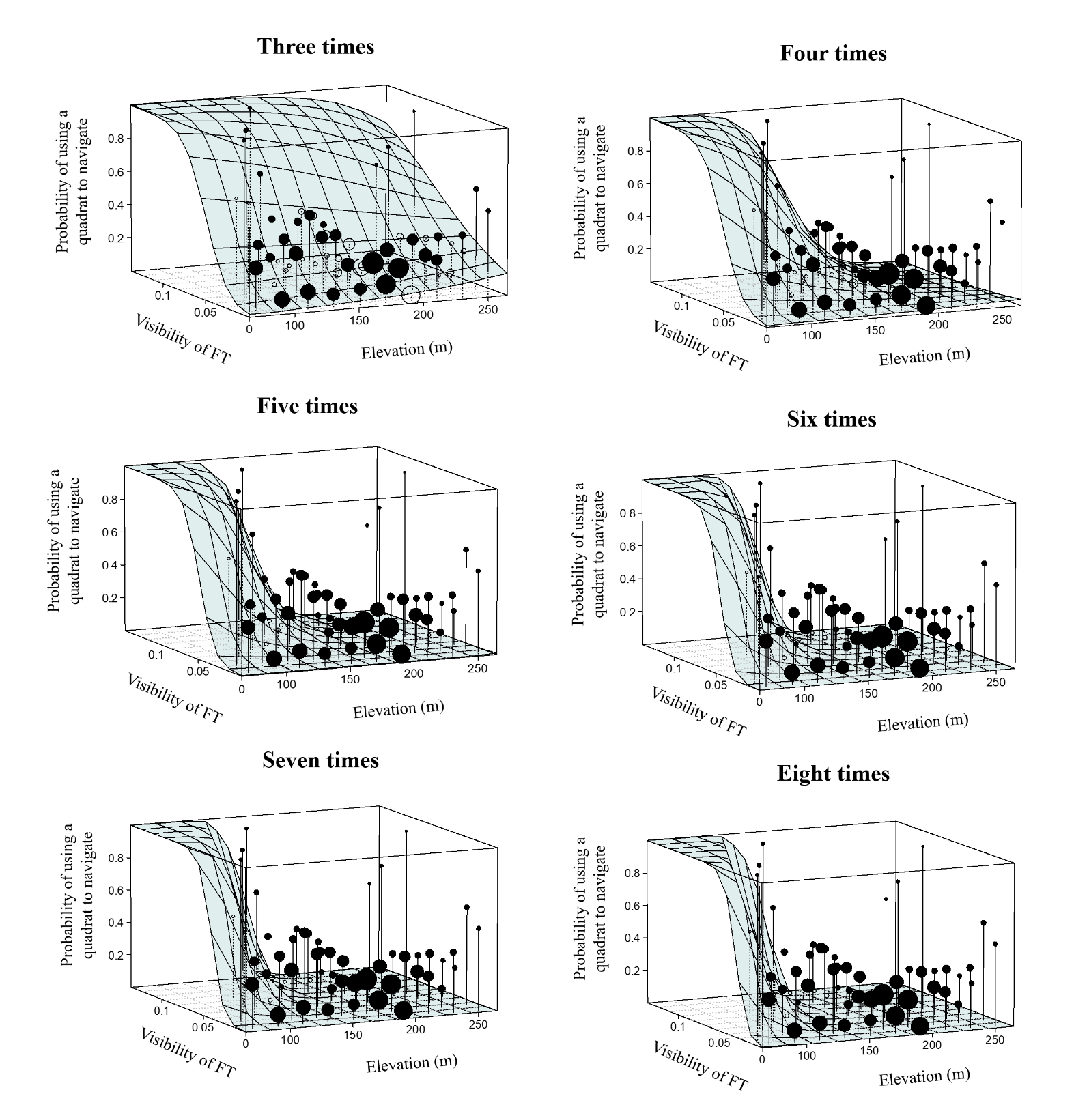
**Fig. S3** Probability that black howler monkeys selected a quadrat to navigate in between three and height different occasions in relation to the relative elevation within their home range and the visibility of FT. The interaction was not significant for routes used at least three times but was always significant for routes used more than three times. The height of spheres represents the probability that a certain quadrat was chosen to navigate per combination of elevation and visibility of FT. Each surface (i.e. square) represents the expected probability of a quadrat to be chosen according to the model (conditional on all other predictors being set at their average value). Sphere size corresponds to the relative number of observations, with closed circles being above the model surface and open circles below.
